# GnRH Induces ERK-Dependent Bleb Formation in Gonadotrope Cells, Involving Recruitment of Members of a GnRH Receptor-Associated Signalosome to the Blebs

**DOI:** 10.3389/fendo.2017.00113

**Published:** 2017-06-02

**Authors:** Liat Rahamim-Ben Navi, Anna Tsukerman, Alona Feldman, Philippa Melamed, Melanija Tomić, Stanko S. Stojilkovic, Ulrich Boehm, Rony Seger, Zvi Naor

**Affiliations:** ^1^Department of Biochemistry and Molecular Biology, Tel Aviv University, Tel Aviv, Israel; ^2^Faculty of Biology, Technion-Israel Institute of Technology, Haifa, Israel; ^3^National Institute of Child Health and Human Development, National Institute of Health, Bethesda, MD, United States; ^4^Department of Pharmacology and Toxicology, University of Saarland School of Medicine, Homburg, Germany; ^5^Department of Biological Regulation, Weizmann Institute of Science, Rehovot, Israel

**Keywords:** GnRH, GnRH receptor, ERK, gonadotropes, blebs, signalosome

## Abstract

We have previously described a signaling complex (signalosome) associated with the GnRH receptor (GnRHR). We now report that GnRH induces bleb formation in the gonadotrope-derived LβT2 cells. The blebs appear within ~2 min at a turnover rate of ~2–3 blebs/min and last for at least 90 min. Formation of the blebs requires active ERK1/2 and RhoA–ROCK but not active c-Src. Although the following ligands stimulate ERK1/2 in LβT2 cells: EGF > GnRH > PMA > cyclic adenosine monophosphate (cAMP), they produced little or no effect on bleb formation as compared to the robust effect of GnRH (GnRH > PMA > cAMP > EGF), indicating that ERK1/2 is required but not sufficient for bleb formation possibly due to compartmentalization. Members of the above mentioned signalosome are recruited to the blebs, some during bleb formation (GnRHR, c-Src, ERK1/2, focal adhesion kinase, paxillin, and tubulin), and some during bleb retraction (vinculin), while F-actin decorates the blebs during retraction. Fluorescence intensity measurements for the above proteins across the cells showed higher intensity in the blebs vs. intracellular area. Moreover, GnRH induces blebs in primary cultures of rat pituitary cells and isolated mouse gonadotropes in an ERK1/2-dependent manner. The novel signalosome–bleb pathway suggests that as with the signalosome, the blebs are apparently involved in cell migration. Hence, we have extended the potential candidates which are involved in the blebs life cycle in general and for the GnRHR in particular.

## Introduction

GnRH interaction with the GnRH receptor (GnRHR) in pituitary gonadotropes is a key step in reproduction (1, 2) (for reviews). The GnRHR is a unique member of the GPCR family, lacking a c-terminal tail (3, 4). The signaling of the GnRHR is complex and includes interaction with heterotrimeric G proteins (G-proteins) primarily *via* the Gq and/or G_11_ (5), stimulation of cyclic adenosine monophosphate (cAMP), protein kinase A, prostaglandins (PGs) (2), Ca^2+^-calmodulin (6–8), protein kinase C isoforms (PKCs), and mitogen-activated protein kinases (MAPKs) (2, 9). The signaling pathways culminate in luteinizing hormone (LH) and follicle-stimulating hormone synthesis and release (1–9).

Mitogen-activated protein kinase cascades in mammals include ERK1/2 (p42 and p44), JNK1/3, p38 (α, β, γ, δ), and ERK5 (10, 11). MAPKs act by sequential phosphorylation and activation of their kinase components (10, 11). MAPKs translocate to the nucleus and activate transcription factors; however, they can also reside and act in the cytosol (10, 11). MAPKs participate in GnRH-induced transcriptional control of the gonadotropin subunits and the GnRHR genes (2, 12–28).

GnRH receptor-associated protein–protein complexes and actin cytoskeletal remodeling events have been described (29–32). We have previously demonstrated the presence of such a complex (signalosome) that seems to reside in microtubules and focal adhesions (FAs) (33). Members of the signalosome included the GnRHR, Ras–MEK–ERK, PKCs, focal adhesion kinase (FAK), paxillin, vinculin, and tubulin (Figure S1 in Supplementary Material). We have proposed that the role of the signalosome is to sequester a pool of GnRH-activated ERK1/2 in the cytosol for the phosphorylation of FAK and paxillin at FAs, to mediate cell migration, as recently proposed for GnRH-stimulated gonadotropes (34, 35).

Cell membrane blebs are dynamic protrusions that are implicated in apoptosis, cytokinesis, and cell movement (36). The blebs are formed by depolymerization of the actin cortex, which leads to rapid bleb formation as a result of the cell internal hydrostatic pressure (36). Blebs expand up to 2 µm from the cell membrane and are defined by a spherical morphology (36). Blebs have highly dynamic life cycle that roughly lasts 1–2 min; rapid bleb expansion, a short static phase; and retraction of the blebs (36–39). Initial expansion of the blebs does not involve actin polymerization, which distinguishes plasma membrane bleb from all other known cell protrusions such as lamellipodia and filopodia (36–39). Actin is subsequently polymerized at the bleb cortex to halt bleb expansion and actomyosin contractility is generated to retract the blebs (40). The contractility for bleb retraction is provided by signaling through Rho-ROCK-myosin. In this cascade, Rho-GTP activates its effector kinase Rho-associated kinase (ROCK) that directly phosphorylates myosin light chain, which then induces actomyosin contraction (36, 41).

Here, we show that GnRH induces bleb formation in the immortalized LβT2 pituitary gonadotrope cells, a process requiring active ERK1/2 and Rho-ROCK but not active c-Src. Members of the above described signalosome are also present in the blebs during bleb formation, stabilization, or retraction, suggesting that they were recruited separately to the blebs. We also confirmed the findings in rat- and mouse-isolated gonadotropes. Hence, we have extended the potential candidates which are involved in the blebs life cycle in general and the GnRHR in particular.

## Materials and Methods

### Materials

Medium, serum, and antibiotics for cell cultures are from Biological Industries (Kibbutz Beit Ha’Emek, Israel). GnRH and PMA were obtained from Sigma (St. Louis, MO, USA). EGF was purchased from Prospec (East Brunswick, NJ, USA). U0126, SB203580, 8-Br-cAMP, mouse monoclonal anti-doubly phosphorylated-ERK1/2 antibodies, and rabbit polyclonal antibodies to general ERK were obtained from Sigma-Aldrich (Rehovot, Israel). jetPRIME Transfection reagent was obtained from polyplus transfection (Illkirch, France). GnRH antagonist (cetrorelix acetate) was from Merck (NJ, USA). The ROCK inhibitor Y-27632 was from Cayman Chemical Company (Ann Arbor, MI, USA). Secondary horseradish peroxidase-conjugated goat anti mouse antibodies or goats anti rabbit antibodies were purchased from Jackson ImmunoResearch Laboratories (West Grove, PA, USA). GFP-ERK2 and c-Src-GFP were kindly provided by Dr. Rony Seger, and Vinculin-GFP was kindly provided by Dr. Benny Geiger (Weizmann Institute of Science, Rehovot, Israel). GnRHR-mCherry construct was kindly provided by Dr. Colin Clay (Colorado State University, USA). Paxillin-GFP and FAK-GFP were kindly provided by Dr. Kenneth Yamada (NIH, USA). Actin-YFP was kindly provided by Dr. Ilan Tsarfaty, and EMTB-3XGFP was kindly provided by Dr. David Sprinzak (Tel-Aviv University, Israel).

### Cell Culture

LβT2 cells (kindly provided by Prof. P. Mellon UCSD, USA) were grown in DMEM supplemented with 10% FCS, streptomycin (100 µg/ml), penicillin (100 U/ml), and 5% glutamine. Cells were maintained in humidified atmosphere of 5% CO_2_ and at 37°C. At 70–80% confluence, the cells were serum starved overnight in DMEM with 0.1% FCS, and stimulants were added in DMEM. Cells were washed twice with ice-cold PBS and overlaid with lysis buffer (20 mM Tris–HCl pH 7.5, 20 mM NaCl, 5 mM MgCl_2_, 1 mM Na_3_VO_4_, 0.5% Tryton x-100, 50 mM β-glycerophosphate, 30% glycerol, 1 mM benzamidine, 10 µg/ml aprotinin, 10 µg/ml leupeptin, 1 mM PMSF), followed by centrifugation (15,000 × *g*, 15 min, 4°C). The supernatants were collected, and aliquots were separated on 10% SDS-PAGE, followed by Western blotting.

### Live Cell Imaging

LβT2 cells were plated on 35 mm glass-bottom plates and were transfected with 1 µg of GnRHR-mCherry along with 1 µg of one of the complex proteins constructs (ERK2–GFP, FAK–GFP, etc.) by using the jet PRIME™ transfection reagent. Approximately 30 h after transfection, the cells were serum starved (0.1% FCS) for 16 h and later stimulated with various ligands and inhibitors as indicated. Images were acquired at 10-s intervals using Leica TCS STED microscope (Leica, Wetzlar, Germany) using the 63 objective. Cells were kept in a microscope stage incubator at 37°C in a humidified atmosphere of 5% CO_2_ throughout the experiment. Data and image analysis was performed using ImageJ (NIH, Bethesda, MD, USA).

### Cell Migration Assay

LβT2 cells (1 × 10^5^) were trypsinized and resuspended in starvation medium (0.1% FCS) and plated in Matrigel (1:150 dilution)-coated transwell inserts with or without the MEK inhibitor, U0126 (25 µM). Lower chambers contained starvation medium with 10 nm GnRH. After 24 h, cells were fixed in 2.5% glutaraldehyde for 15 min and washed with DDW. Cells were stained with 0.1% methylene blue for 60 min. Cells that did not migrate to the underside of the membrane were scraped off using a cotton swab. Migrated cells were observed under a microscope and counted from ten random fields.

### Primary Culture of Anterior Pituitary Cells

Post-pubertal female Sprague-Dawley rats obtained from Taconic Farms (Germantown, NY, USA) were euthanized by asphyxiation with CO_2_, and the anterior pituitary glands were removed after decapitation. The procedure was approved by the NICHD Animal Care and Use Committee (#14-041). The methods were carried out in accordance with the approved guidelines. Pituitary tissue was cut into 1 mm^3^ pieces, treated with trypsin (20 µg/ml diluted in PBS + 0.3% BSA medium) for 15 min at 37°C, and followed by addition of a pinch of DNAase and 2.6 mg/ml trypsin inhibitor. Mechanical dispersion of cells was done in calcium-deficient PBS medium. Dispersed anterior pituitary cells were plated on poly-l-lysine coated 25 mm circular coverslips (Thomas Scientific, Swedesboro, NJ, USA) at 7 × 10^5^ cells/coverslip density and cultured overnight in medium-199 containing Earle’s salts and supplemented with 10% horse serum, penicillin (100 U/ml), and streptomycin (100 µg/ml) (Life Technologies). At least an hour prior to experiments, the medium was changed to Krebs-Ringer containing 2.5 µM Fura-2 AM (Life Technologies). The coverslips were then washed in Krebs-Ringer medium and mounted on the stage of an inverted Observer-D1 microscope (Carl Zeiss, Oberkochen, Germany) with an attached ORCA-ER camera (Hamamatsu Photonics, Hamamatsu City, Japan) and a Lambda DG-4 wavelength switcher (Sutter, Novato, CA, USA). Hardware control and image analysis was performed using Metafluor software (Molecular Devices, Downingtown, PA, USA). Experiments were performed with a 63× oil-immersion objective while alternatively recording transmitted light image and the image at 380 nm excitation beam. There were approximately 20 mixed pituitary cells in the field, and the gonadotropes were identified by their intracellular Ca^2+^ response to GnRH, i.e., rapid decrease of 380 nm-induced fluorescence intensity, followed by a slower increase. After that, blebbing was analyzed. The images were further analyzed using ImageJ (NIH, Bethesda, MD, USA).

### Preparation of Primary Gonadotrope Cultures from GRIC-Ai9 Mice

In order to confirm our findings from the gonadotrope-derived cell line, we have prepared primary gonadotropes from transgenic mice that carry a fluorescent signal in their gonadotropes, the GRIC/Ai9 mice. GRIC mice express Cre recombinase driven by the promoter of the GnRHR gene. Therefore, when Ai9 mice are crossed with GRIC mice the stop cassette is excised, which activates constitutive expression of dTomato in the gonadotropes. These mice thus allow identification and sorting of the gonadotrope cells_._ Animals were held and handled after protocol approval by the Technion IACUC and in accordance with their guidelines and regulations. We prepared primary gonadotrope culture from heterozygous mice created by breeding GRIC females with Ai9 males. Sexually mature female heterozygous mice were sacrificed, their pituitaries removed, and pituitary cells were prepared as previously described (42, 43). The gonadotropes were collected from the total pituitary population based on their fluorescence, using a FACS Aria 2 sorter. Following sorting, the cells were plated on glass-bottom plates for 12 h in fresh medium (DMEM 10% FCS). At least an hour prior to experiments, cells were serum starved (0.1% FCS), later stimulated with various ligands and inhibitors. Images were acquired using Leica TCS STED microscope (Leica, Wetzlar, Germany) using the 63 objective. Cells were kept in a microscope stage incubator at 37°C in a humidified atmosphere of 5% CO_2_ throughout the experiment. Data and image analysis was performed using ImageJ (NIH, Bethesda, MD, USA).

### Statistical Analysis

Results from three or more experiments were expressed as mean ± SEM. Where appropriate, data were subjected to statistical analysis by Student’s *t*-test, or by one- or two-way ANOVA, depending on the experimental design. Values of *p* < 0.05 were considered statistically significant.

## Results

### GnRH Induces Bleb Formation and GnRHR Is Present in the Blebs

**Figure 1:**
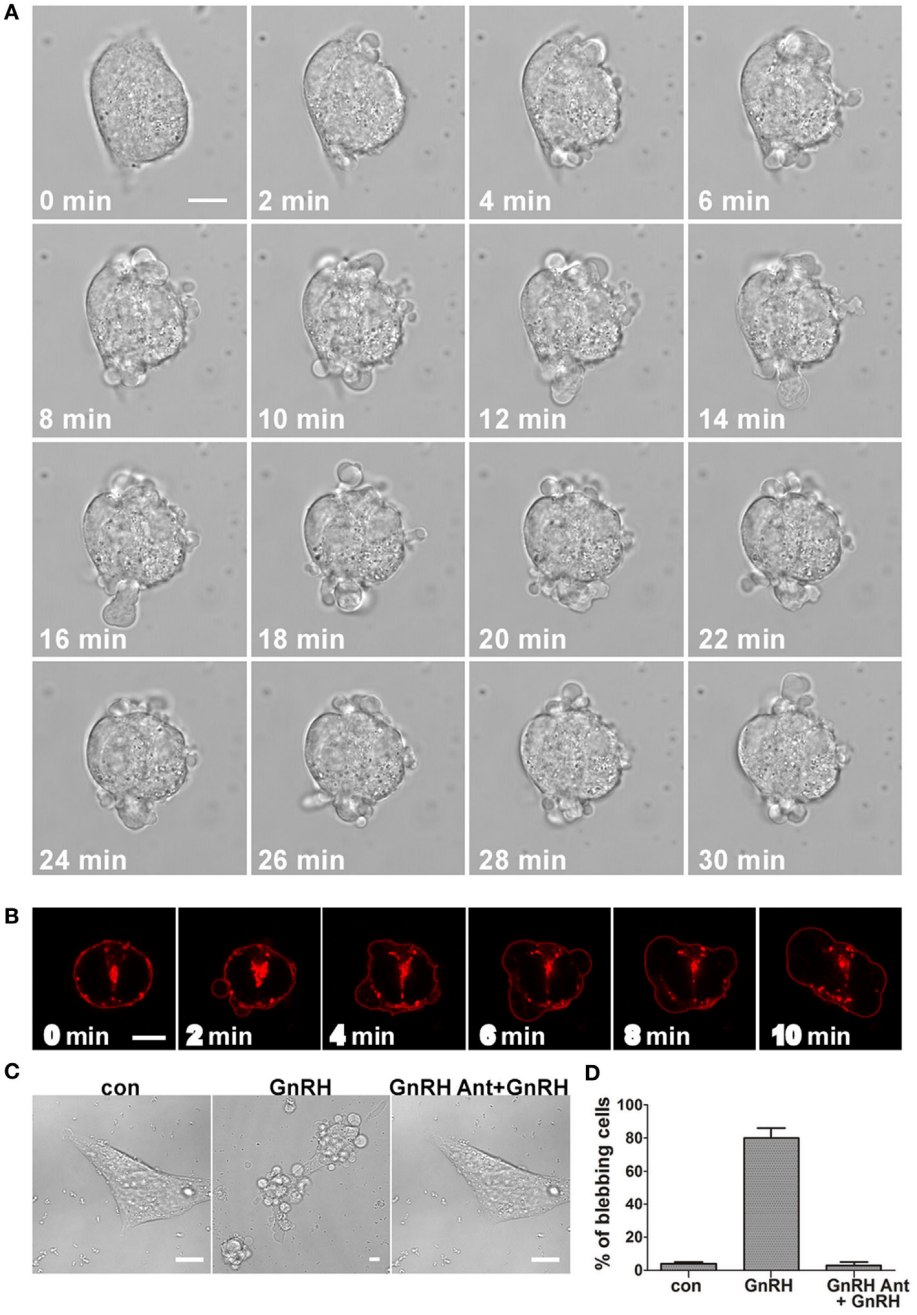
**GnRH induces bleb formation in LβT2 cells and GnRH receptor (GnRHR) is present in the blebs**. **(A)** Images from a confocal microscopy time-lapse movie of serum-starved LβT2 gonadotrope cells treated with GnRH (10 nM) (0–30 min). The treatment resulted in bleb formation. The *scale bar* is 10 µm. **(B)** GnRHR is present in the blebs. Serum-starved LβT2 cells were subjected to time laps confocal microscopy. Addition of GnRH (10 nM) (0–10 min) to LβT2 cells transfected with GnRHR-mCherry resulted in bleb formation, while GnRHR is present in the blebs. The *scale bar* is 5 µm. **(C)** GnRHR mediates the formation of the blebs by GnRH. Serum-starved LβT2 cells were incubated with GnRH (10 nM) (30 min), or preincubated first with 100 nM GnRH antagonist (cetrorelix acetate) for 30 min followed by GnRH (10 nM) (GnRH antagonist + GnRH) for additional 30 min. Cells were imaged using confocal microscope and *the scale bar* is 10 µm. **(D)** Quantitation of blebbing cells in control, GnRH and GnRH antagonist + GnRH. Images of at least 10 fields were taken for each treatment, and the *bars* are mean ± SEM from 3 experiments.

**Figure 2:**
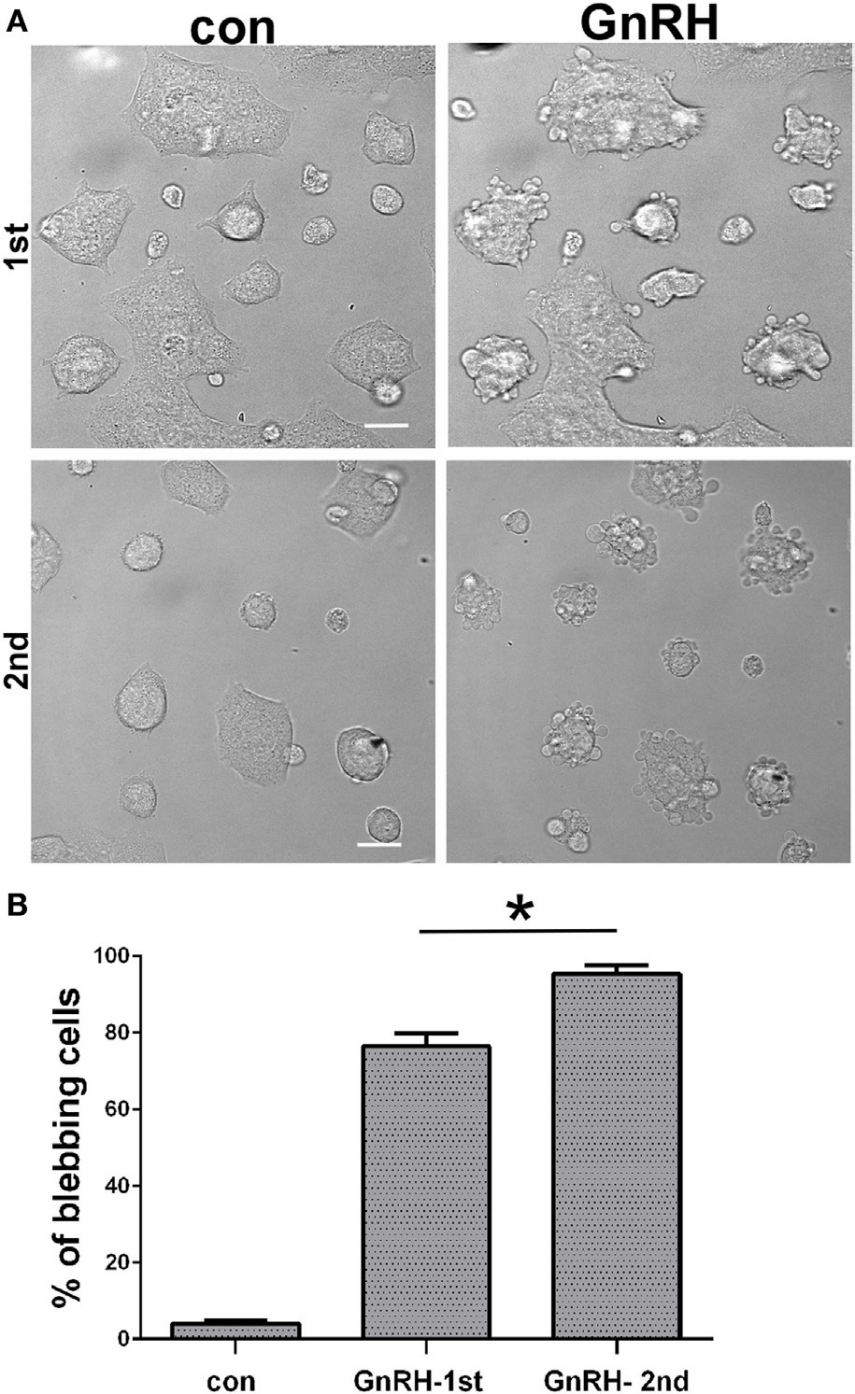
**Priming effect of GnRH upon blebs formation**. **(A)** Serum-starved LβT2 cells were incubated for 30 min with 10 nM GnRH and analyzed for blebs formation as above. Another group of cells were first incubated for 30 min with 10 nM GnRH, at the end of which GnRH was removed by a wash. Six hours later, a second dose of 10 nM of GnRH was added to the cells. Both groups of cells were imaged using confocal microscopy. The *scale bar* is 20 µm. **(B)** Quantitation of blebbing cells in control, GnRH (first treatment) and GnRH (second treatment). Images of at least 10 fields were taken for each treatment, and the *bars* are mean ± SEM. *p-Value ≤0.05 vs. GnRH first exposure.

Time-lapse confocal microscopy of GnRH-treated LβT2 cells showed that GnRH induces bleb formation (Figure 1A) (see also Video S1 in Supplementary Material). The blebs appear within ~2 min and last for at least 90 min at the apparent turnover rate of ~2–3 blebs/min. In order to further investigate the involvement of the GnRHR in bleb formation, LβT2 cells were transfected with GnRHR-mCherry and then treated with GnRH for 30 min. Under basal conditions, GnRHR was observed in the membrane (Figure 1B), while after GnRH treatment, GnRHR decorated the blebs membrane, with no difference between blebs expansion and retraction. Preincubation with the GnRH antagonist (cetrorelix acetate), abolished bleb formation by GnRH (Figures 1C,D), confirming that bleb formation is mediated by the GnRHR. In addition, the cells returned to pretreatment morphology after removal of GnRH indicating that the process is reversible. Retreatment with GnRH (30 min) 6 h later resulted in a “priming effect,” which is defined as an increase in cells response to the second exposure to GnRH compared with the first. Indeed, the second exposure to GnRH elevated the percentage of blebbing cells (Figure 2).

### ERK1/2 Accumulates in the Blebs and Is Involved in Bleb Formation

**Figure 3:**
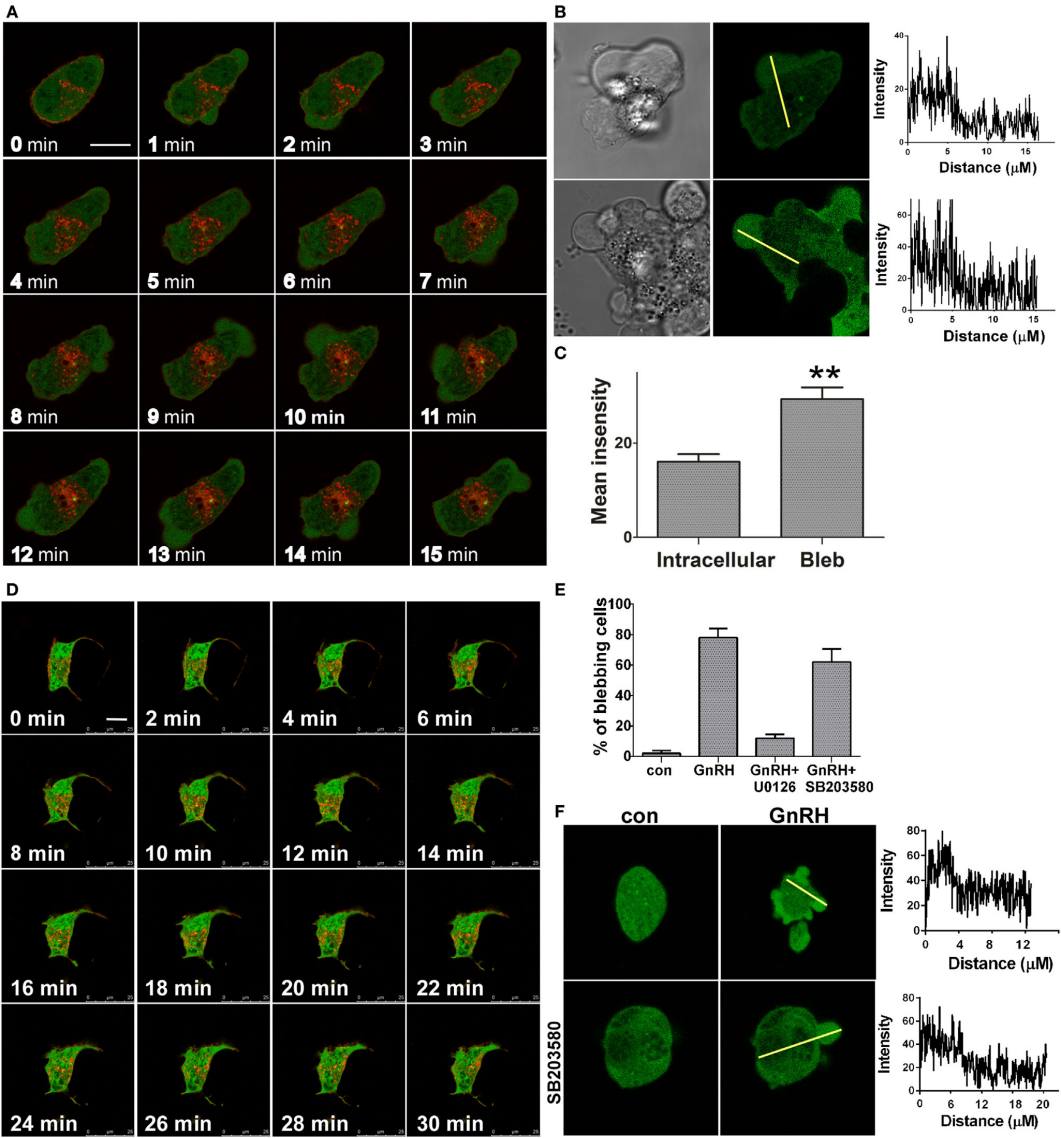
**ERK1/2 is present and involved in bleb formation**. **(A)** Addition of GnRH (0–15 min, 10 nM) to serum-starved LβT2 cells transfected with GnRH receptor (GnRHR)-mCherry and ERK-GFP resulted in bleb formation, while ERK1/2 accumulates in the blebs. The *scale bar* is 10 µm. **(B)** Blebs images after GnRH treatment including differential interference contrast (DIC) and fluorescent images of ERK2-GFP. A line intensity profile across the cells was obtained and intensity profiles are shown on the right. **(C)** Bars show mean ± SEM of fluorescence intensity of the blebs vs. intracellular area from multiple scanning of each cell from at least five experiments. **p-Value ≤0.01. **(D)** Addition of the MEK selective inhibitor U0126 (25 µM) 20 min prior to GnRH (0–30 min, 10 nM) to serum-starved LβT2 gonadotrope cells transfected with GnRHR-mCherry and ERK2-GFP abolished bleb formation. Similar results were observed in two other experiments. The *scale bar* is 10 µm. **(E)** ERK1/2, but not p38MAPK, is involved in bleb formation. Serum-starved LβT2 gonadotrope cells were pretreated with U0126 or SB203580 (MEK and p38MAPK selective inhibitors, respectively) at 25 µM for 20 min prior to GnRH (10 nM, 30 min). Quantitation of the percentage of blebbing cells is shown. Images of at least 10 fields were taken for each treatment, and the *bars* are mean ± SEM from 3 experiments. **(F)** Pretreatment with SB203580 did not attenuate GnRH-induced ERK1/2 accumulation in the blebs as indicated by fluorescence intensity measurements. Serum-starved LβT2 cells transfected with ERK-GFP and were pretreated with or without SB203580 (25 µM) for 20 min prior to GnRH (10 nM, 30 min). A line intensity profile across the cells was obtained, and intensity profiles are shown on the right.

ERK1/2 activation by GnRH in LβT2 cells was reported to involve PKC, Ca^2+^ influx, dynamin, and c-Src (20, 44, 45). Previous studies in our laboratory have examined the kinetics of ERK1/2 activation in response to GnRH treatment in LβT2 cells. GnRH treatment resulted in a rapid and robust activation of ERK1/2 with a peak 5 min after stimulation and decline but still detectable after 90 min (44). It is thought that RTK and GPCR ligands induce a rapid translocation of ERK1/2 to the nucleus to phosphorylate and activate transcription factors (10, 11). In order to understand the involvement of ERK1/2 in bleb formation, we followed the cellular localization of ERK1/2 in response to GnRH treatment. LβT2 cells were transfected with GnRHR-mCherry and ERK2-GFP and then treated with GnRH for 15 min. Time-lapse confocal microscopy showed that GnRH-induced ERK1/2 accumulation in the blebs within 1 min (Figure 3A) (see also Video S2 in Supplementary Material). A line intensity profile across the cell was obtained (Figure 3B) and intensity profiles shown on the right demonstrate accumulation of ERK1/2 in the blebs. Quantitation of mean fluorescence intensity showed higher values of ERK1/2 in the blebs vs. intracellular area (without the blebs area) (Figure 3C). Moreover, preincubation with the MEK inhibitor U0126 strongly inhibits GnRH-induced bleb formation (Figure 3D). Also, preincubation with the MEK inhibitor U0126 strongly reduced GnRH-induced cell migration (data not shown) suggesting that the bleb formation may be involved in cell migration. Pretreatment with SB203580, a p38 inhibitor did not attenuate the bleb formation (Figure 3E). Quantitation confirmed that compared to GnRH treated cells, U0126 reduced cell blebbing and SB203580 had no significant effect (Figure 3E). In addition, fluorescence intensity was measured across the cells and the profiles shown in line graphs on the right indicates that ERK2 accumulates in the blebs even in the presence of the p38 inhibitor (Figure 3F).

### ERK1/2 Activation Is Required, but Not Sufficient for Bleb Formation

**Figure 4:**
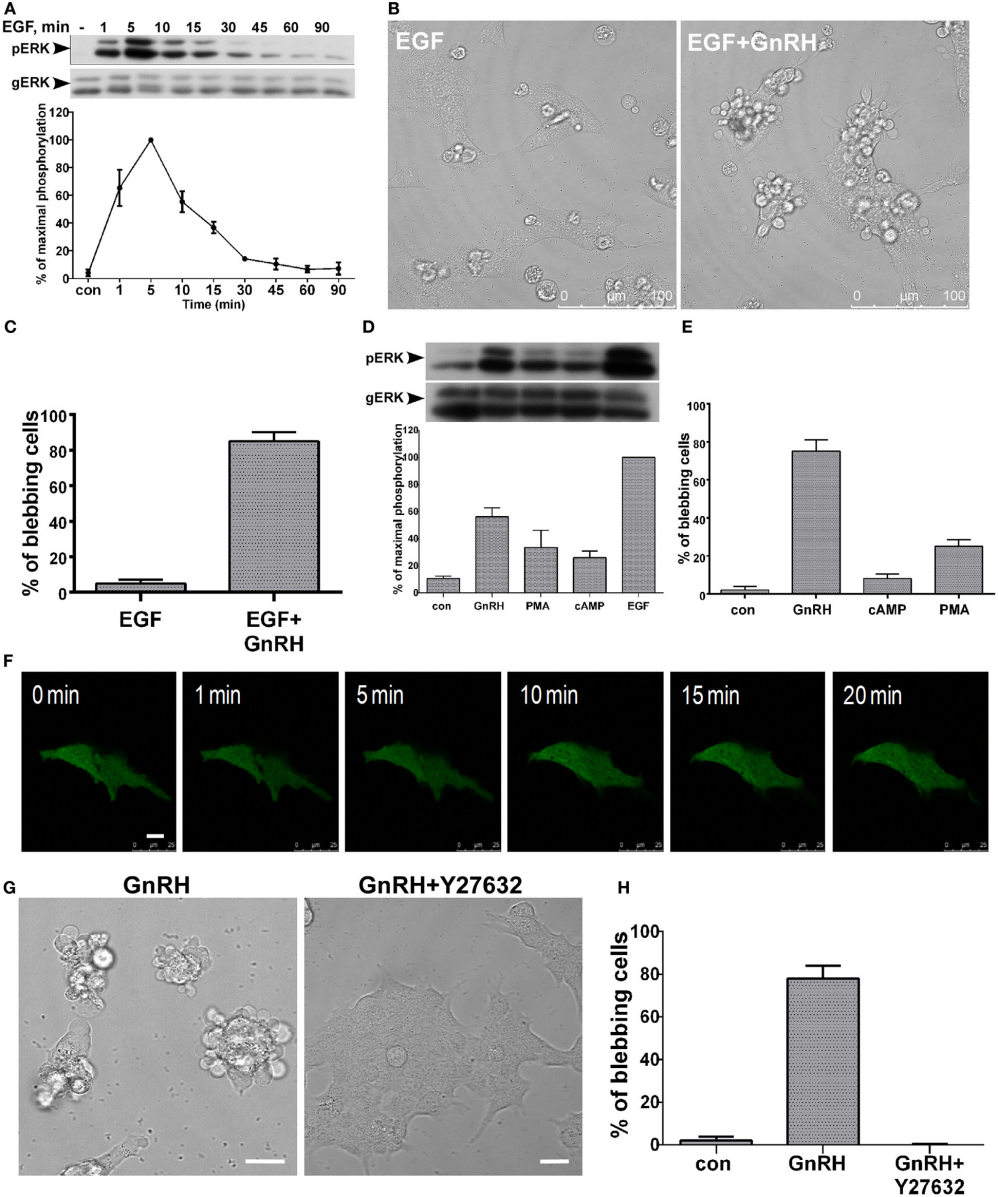
**(A–E)** ERK1/2 activation is required, but not sufficient for bleb formation. **(A)** Serum-starved LβT2 were treated with epidermal growth factor (EGF) (10 ng/ml, 0–90 min). Thereafter, cell lysates were analyzed for ERK1/2 activity by Western blotting using an antibody for phospho-ERK1/2. Total ERK (gERK) was detected with polyclonal antibody as a control for sample loading. Results are shown as mean ± SEM of maximal phosphorylation. A representative blot is shown and similar results were observed in two other experiments. **(B)** Addition of EGF (10 ng/ml, 30 min) to LβT2 cell resulted in minimal bleb formation. Then, addition of GnRH (30 min, 10 nM) resulted in marked elevation of bleb formation. **(C)** Quantitation of blebbing cells in EGF and EGF + GnRH treatment are shown. Images of at least 10 fields were taken for each treatment, and the *bars* are mean ± SEM from 3 experiments. **(D)** Serum-starved LβT2 gonadotrope cells were treated with GnRH (10 nM), PMA (50 nM), 8-Br-cAMP (1 mM), or EGF (10 ng/ml) for 5 min. Cell lysates were analyzed for ERK2 activity by Western blotting using an antibody for phospho-ERK1/2. Total ERK (gERK) was detected with a polyclonal antibody as a control for sample loading. Results are shown as mean ± SEM of maximal phosphorylation from three experiments. **(E)** Quantitation of the percentage of blebbing cells after GnRH (10 nM), PMA (50 nM), or 8-Br-cAMP (1 mM) treatment (30 min). Images of at least 10 fields were taken for each treatment, and the *bars* are mean ± SEM from 3 experiments. **(F–H)** RhoA–ROCK are involved in GnRH-induced bleb formation. **(F)** LβT2 gonadotrope cells were transfected with ERK2-GFP, while 30 h after transfection cells were serum-starved, and preincubated with Y-27632 (a ROCK selective inhibitor, 10 µM) for 20 min prior to GnRH (20 min, 10 nM). Y-27632 abolished bleb formation. The *scale bar* is 10 µm. **(G)** Images of DIC from a confocal microscope of LβT2 gonadotrope cells. Serum-starved LβT2 cells were pretreated with or without Y-27632 (10 µM) for 20 min prior to GnRH (30 min, 10 nM). The *scale bar* is 20 µm. **(H)** Quantitation of blebbing cells in control, GnRH, and GnRH + Y-27632 (added 20 min before GnRH) treatment as in panel **(G)**. Images of at least 10 fields were taken for each treatment, and the *bars* are mean ± SEM from 3 experiments.

Epidermal growth factor (EGF) plays important roles in proliferation, differentiation, and migration *via* stimulation of the ERK1/2 signaling pathway (46). Moreover, Bonfil et al. (20) reported that ERK1/2 activation by GnRH in LβT2 cells is mediated by PKC, Ca^2+^ influx, dynamin, and c-Src, and not *via* transactivation of the EGFR. PMA is a PKC activator, which mimics the action of the naturally occurring DAG by binding to the C1 region of PKC, thus activating the enzyme (47–49). PMA mimicked the activation of ERK1/2 by GnRH (44). In addition, GnRH stimulates cAMP production in LβT2 gonadotrope cells *via* PKCδ (50). We therefore examined the effect of the various ligands on ERK1/2 activation and bleb formation since we have shown above that active ERK1/2 is required for bleb formation (Figure 3). Addition of EGF resulted in rapid activation of ERK1/2 with a peak after 5 min, similar to the effect of GnRH (33) (Figure 4A). LβT2 cells were treated with EGF for 30 min, and time-lapse confocal microscopy revealed minimal bleb formation (Figure 4B). GnRH was then added to the EGF-pretreated cells and significant elevation of bleb formation was observed (Figures 4B,C). The ligands induced ERK1/2 activation in the rank order of: EGF > GnRH > PMA > cAMP (Figure 4D). Later, we examined the effect of the ligands on bleb formation. cAMP and PMA induced relatively small amount of blebs (Figure 4E). The rank order for bleb formation differs from that obtained for ERK1/2 activation and is: GnRH > PMA > cAMP > EGF (Figures 4C,E). Therefore, we propose that ERK1/2 activation is required, but not sufficient for bleb formation. Furthermore, the data suggest compartmentalization of the ERK1/2 signal to the blebs in a ligand-dependent manner, since GnRH-activated ERK1/2, was preferentially sorted also to the blebs.

### RhoA–ROCK Is Involved in GnRH-Induced Bleb Formation

The Rho family members RhoA, Rac1, and Cdc42 are implicated in actin cytoskeleton rearrangements. Godoy at el. (35) showed that GnRH activates Rho family members and increases cell motility. We therefore examined whether RhoA/ROCK is involved in GnRH-induced bleb formation. Preincubation with the ROCK inhibitor Y27632 abolished GnRH-induced bleb formation (Figures 4F–H). The findings indicate that RhoA–ROCK is involved in GnRH-induced bleb formation.

### c-Src Is Present in the Blebs

**Figure 5:**
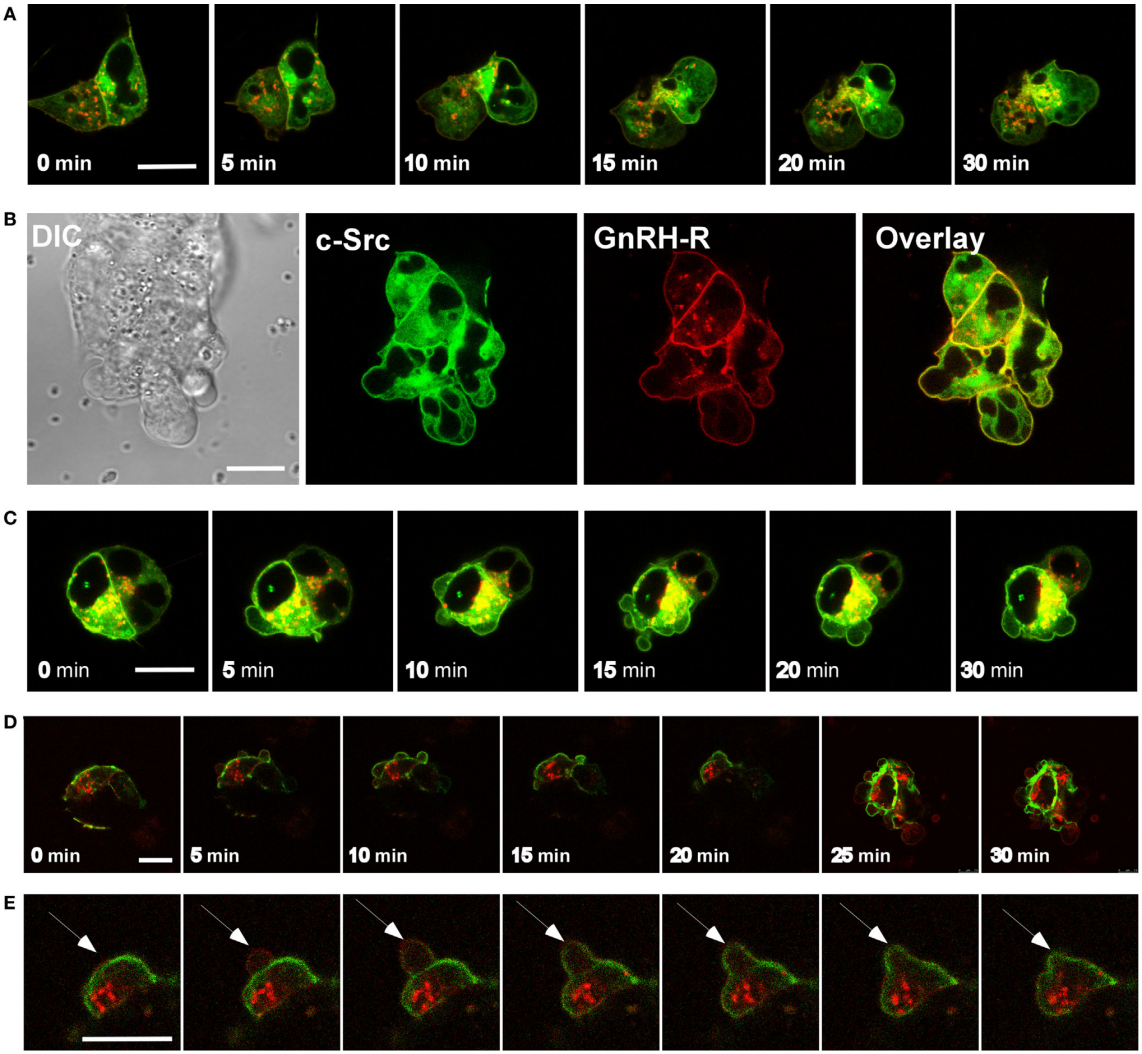
**c-Src and vinculin are present in the blebs**. **(A)** Addition of GnRH (0–30 min, 10 nM) to serum-starved LβT2 cells transfected with c-Src-GFP and GnRH receptor (GnRHR)-mCherry resulted in bleb formation, while c-Src is present in the blebs. The *scale bar* is 10 µm. **(B)** Blebs time laps of DIC and fluorescent images of Src-GFP, GnRHR-mCherry, and overlay showing colocalization of c-Src and the GnRHR in the blebs. The *scale bar* is 10 µm. **(C)** Serum-starved LβT2 cells transfected with c-Src-GFP and GnRHR-mCherry were pretreated with the c-Src inhibitor PP2 (10 µM) for 30 min. Thereafter, GnRH (10 nM) was added for 30 min. Similar results were observed in two other experiments. The *scale bar* is 10 µm. **(D)** Addition of GnRH (0–30 min, 10 nM) to serum-starved LβT2 cells transfected with GnRHR-mCherry and vinculin-GFP resulted in bleb formation, while vinculin is present in the blebs. The *scale bar* is 10 µm. **(E)** Unlike c-Src, ERK1/2, focal adhesion kinase, and paxillin (see below), vinculin was recruited to the blebs during stabilization and retraction. Addition of GnRH (10 nM) to serum-starved LβT2 cells transfected with GnRHR-mCherry and vinculin-GFP resulted in bleb formation, single blebs were monitored every 10 seconds. The *scale bar* is 10 µm.

Our previous studies showed that GnRH activates ERK1/2 in LβT2 gonadotrope cells in a c-Src-dependent manner (20). The modular SH1, SH2, and SH3 and kinase domains of the Src family tyrosine kinases allow these domains to act as scaffolds for diverse signaling proteins (51). Since c-Src is a member of the signalosome (33), we followed its cellular localization in response to GnRH treatment. LβT2 cells were transfected with c-Src-GFP and GnRHR-mCherry and later treated with GnRH for 30 min (Figure 5A). Time-lapse confocal microscopy showed that c-Src is present in the blebs, with no difference between expansion and retraction of the blebs (Figure 5A). Furthermore, the data show the colocalization of c-Src and the GnRHR in the blebs (Figure 5B). Inhibition of c-Src activity by PP2, which we have shown previously (20), had no effect on bleb formation (Figure 5C), suggesting that active c-Src is not required for bleb formation by GnRH. Thereafter, we have chosen to follow other members of the signalosome known to interact with c-Src in relation to their presence in the blebs.

### Vinculin Is Present in the Blebs

Vinculin is a scaffold protein, which binds to actin filament and is localized to FAs (52). Vinculin controls and regulates FA formation and cell migration. It is known that vinculin regulates survival and motility *via* ERK1/2 by controlling the accessibility of paxillin for FAK interaction (53). Since vinculin is a member of the signalosome and binds paxillin, we examined its involvement in bleb formation in response to GnRH treatment by live imaging microscopy. LβT2 cells were transfected with GnRHR-mCherry and vinculin-GFP and then treated with GnRH for 30 min. Time-lapse confocal microscopy showed that vinculin accumulates in the blebs (Figure 5D). However, unlike ERK1/2, c-Src, FAK, and paxillin (see below), vinculin was not present in the initial bleb expansion and was detected after the blebs were stabilized (Figure 5E). Therefore, we assume that vinculin is involved in bleb retraction.

### FAK Is Present in the Blebs

**Figure 6:**
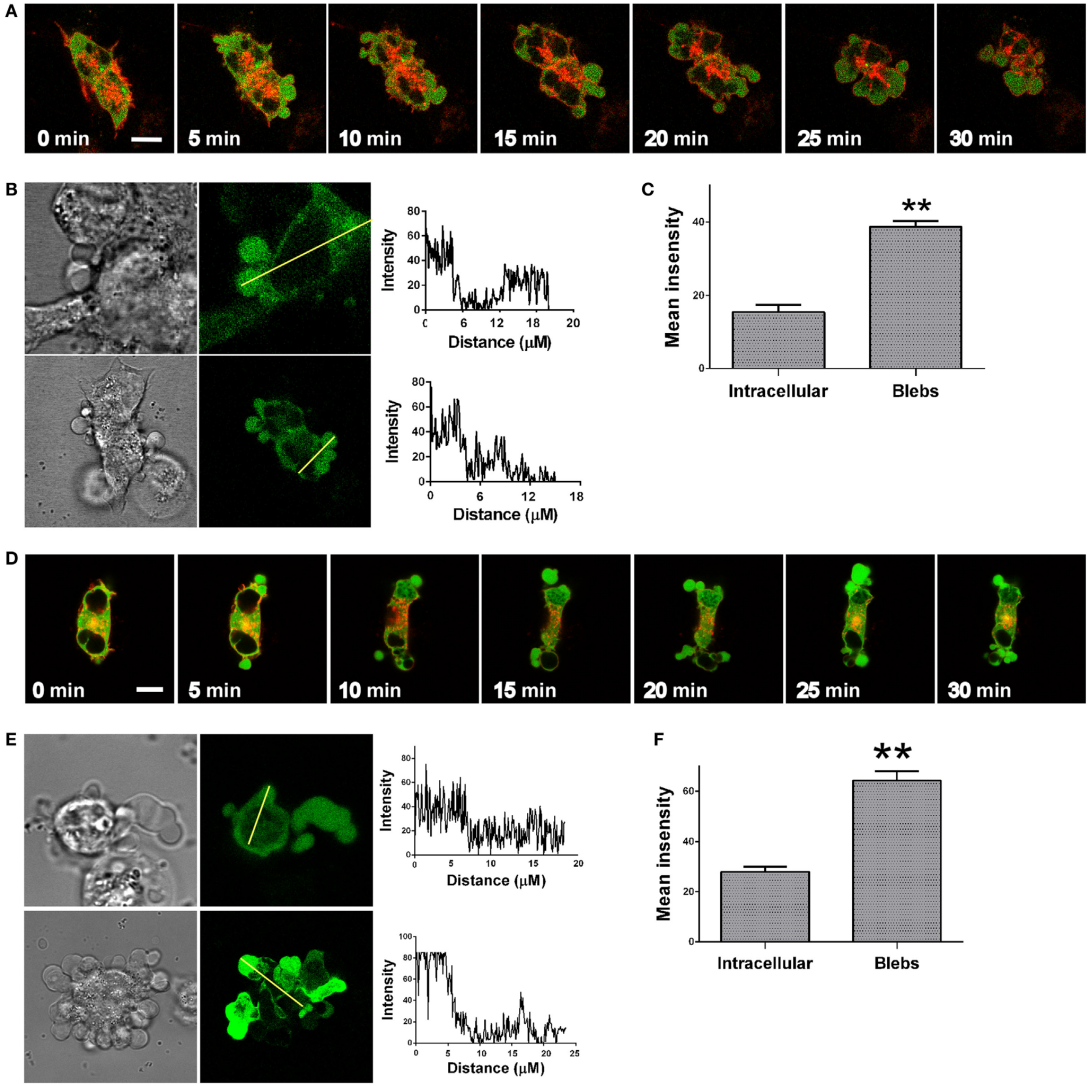
**Focal adhesion kinase (FAK) and paxillin are present in the blebs**. **(A)** Addition of GnRH (0–30 min, 10 nM) to serum-starved LβT2 cells transfected with GnRH receptor (GnRHR)-mCherry and FAK-GFP resulted in bleb formation, while FAK is present in the blebs. The *scale bar* is 10 µm. **(B)** Blebs images after GnRH treatment, including DIC and fluorescent images of FAK-GFP. Fluorescence intensity was measured across the cells and intensity profiles are shown on the right. **(C)** Quantitation of FAK-GFP mean fluorescence intensity in the blebs vs. intracellular area from multiple scanning of each cell from at least five experiments. The *bars* are mean ± SEM. **p-Value ≤0.01. **(D)** Addition of GnRH (30 min, 10 nM) to serum-starved LβT2 cells transfected with GnRHR-mCherry and paxillin-GFP resulted in bleb formation, while paxillin accumulates in the blebs. The *scale bar* is 10 µm. **(E)** Blebs images after GnRH treatment, including DIC and fluorescent images of paxillin-GFP. A line intensity profile across the cells was obtained and intensity profiles are shown on the right. **(F)** Histograms show mean ± SEM of fluorescence intensity of the blebs vs. intracellular area from multiple scanning of each cell from at least five experiments. **p-Value ≤0.01.

Focal adhesion kinase is a non-receptor cytoplasmic tyrosine kinase that plays a key role in the regulation of proliferation and migration of normal and tumor cells. FAK associates with integrin receptors and recruits a number of SH2- and SH3-domain-containing proteins to the site of this interaction, thus forming a signaling complex that transmits signals from the extracellular matrix to the cell cytoskeleton (54). Since FAK binds c-Src and paxillin and is a member of the signalosome (33), we followed its presence in bleb formation. LβT2 cells were transfected with GnRHR-mCherry and FAK-GFP and then treated with GnRH for 30 min (Figure 6A). Time-lapse confocal microscopy showed that FAK is present in the blebs, while the GnRHR decorates the membrane but most of the receptors are retained in the cells as observed by others (55). Fluorescence intensity was measured across the cells and the profiles shown in line graphs on the right indicate that FAK accumulates in the blebs (Figure 6B). In addition, histograms show higher mean fluorescence intensity in the blebs vs. intracellular area (without the blebs area) (Figure 6C).

### Paxillin Is Present in the Blebs

Paxillin, a multi-domain adapter protein, belongs to the FAs protein family and is known to interact with Ras, c-Src, tubulin, vinculin, and FAK (members of the signalosome) and is targeted to FAs *via* its LIM 1–4 domains (56, 57). It is thought that c-Src phosphorylation of Tyr118 of paxillin creates an ERK1/2-binding site. The activated paxillin then binds to Raf and MEK to activate ERK1/2. Moreover, the interaction with paxillin partially prevents ERK1/2 nuclear translocation, indicating that the task of restricting ERK1/2 in the cytosol is apparently carried out at least in part by paxillin. ERK1/2 phosphorylation of paxillin on Ser/Thr residues facilitates paxillin association with FAK (58, 59). Paxillin, together with FAK, is essential for cell spreading and migration (60, 61) as we have suggested for the signalosome (33). As indicated paxillin binds c-Src, FAK, and ERK1/2 and is a member of the signalosome, and therefore, we followed the cellular localization of paxillin in response to GnRH treatment. LβT2 cells were transfected with GnRHR-mCherry and paxillin-GFP and then treated with GnRH for 30 min (Figure 6D). Time-lapse confocal microscopy showed that paxillin accumulates in the blebs. Intensity profiles across the cells were obtained (Figure 6E), and graphs shown on the right demonstrate accumulation of paxillin in the blebs. Quantitation of mean fluorescence intensity (Figures 6F) shows higher values in the blebs vs. intracellular area (without the blebs area) as described above for FAK.

### α-Tubulin, but Not Microtubules, Is Present in the Blebs

**Figure 7:**
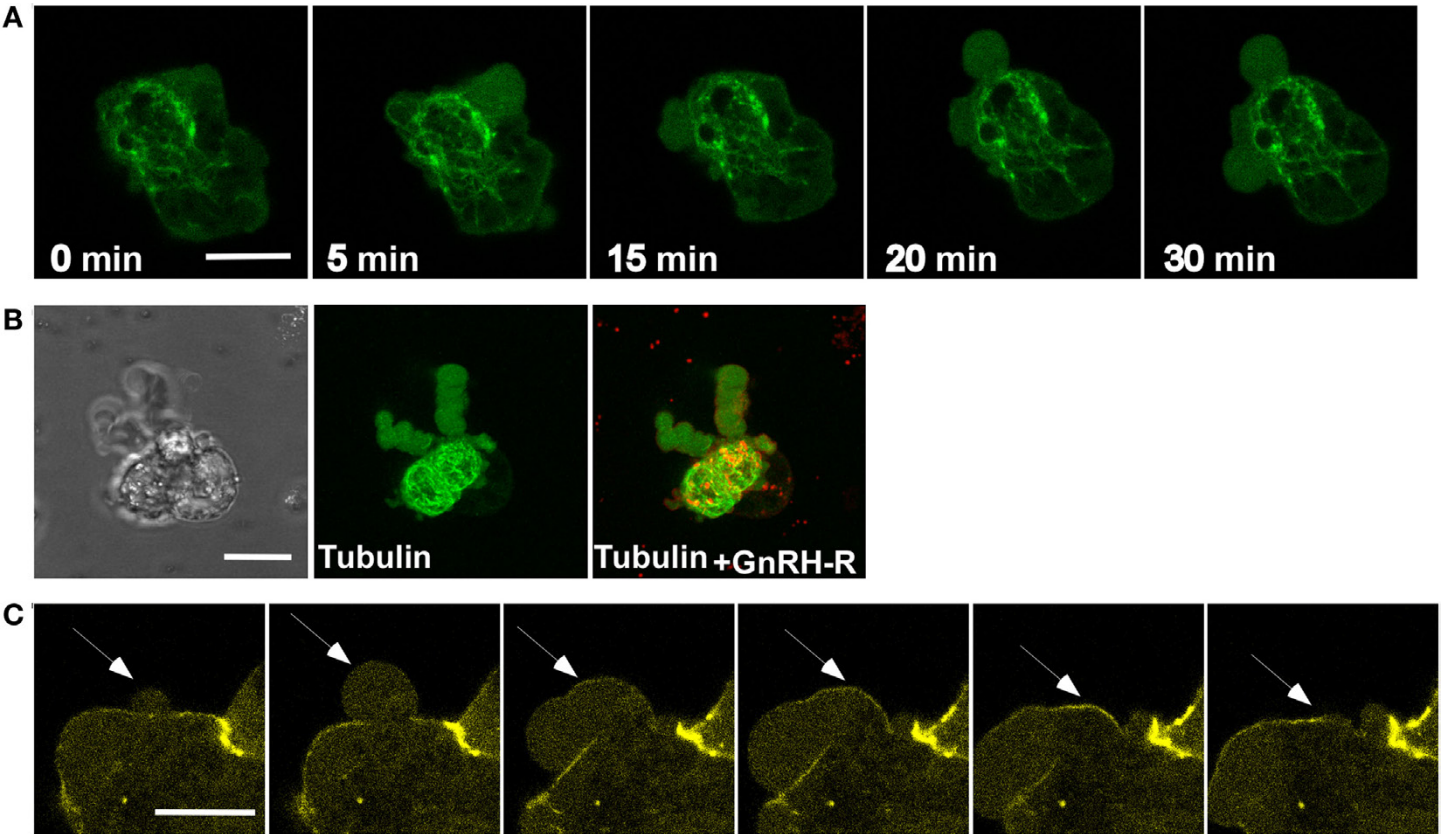
**Tubulin and actin, but not microtubules, are present in the blebs**. **(A)** Images from a confocal microscopy time-lapse movie of serum-starved LβT2 cells transfected with GnRH receptor (GnRHR)-mCherry and EMTB-3XGFP (the microtubule-binding domain of ensconsin (EMTB) fused to three GFP molecules, allowing microtubules visualization) and treated with GnRH (30 min, 10 nM). Bleb formation was noticed, while microtubules are not present in the blebs. The *scale bar* is 10 µm. **(B)** Blebs images after GnRH treatment, including DIC and fluorescent images of GnRHR-mCherry and EMTB-3XGFP, supporting the data observed in panel **(A)**. The *scale bar* is 10 µm. **(C)** Actin is involved in bleb retraction. Addition of GnRH (30 min, 10 nM) to serum-starved LβT2 cells transfected with actin-YFP resulted in bleb formation. Actin is recruited to the blebs after they are stabilized and is best observed during blebs retraction (see arrows). The *scale bar* is 10 µm.

Microtubules are an important part of the cytoskeleton and play a vital role in many cellular processes, such as intracellular transport, mitosis, meiosis, and motility. Microtubules are composed of α–β-tubulin heterodimers (62). Since α-tubulin binds paxillin (57) and ERK1/2 (63) and is a member of the signalosome, we followed its presence in the process of bleb formation. LβT2 cells were transfected with GnRHR-mCherry and EMTB-3XGFP, which is the microtubule binding domain of ensconsin (EMTB) fused to 3GFP molecules, allowing microtubules visualization. Then, cells were treated with GnRH for 30 min. Time-lapse confocal microscopy showed the presence of microtubules fibers in the cells but not in the blebs, while α-tubulin was present in the blebs (Figures 7A,B).

### Actin Is Present in GnRH-Induced Blebs Retraction

The actin cytoskeleton is a central structure for various intracellular processes, such as vesicle transport, cell shape, cell division, motility, cell signaling, and morphogenesis (64). Furthermore, actin depolymerization and polymerization are involved in blebs life cycle (39, 40). Moreover, actin is involved in GnRH to ERK1/2 signaling (30, 34, 65). To examine the present of actin in GnRH-induced bleb formation, LβT2 cells were transfected with actin-YFP and then treated with GnRH for 30 min. Actin is not present in the blebs during blebs expansion. However, we could detect actin at the steady phase of the blebs and during the retraction (Figure 7C). The results are interesting since actin is not present in the signalosome (33), suggesting that blebs member’s proteins are not restricted to those present in the signalosome.

### GnRH Induces ERK1/2-Dependent Bleb Formation in Primary Cultures of Rat Pituitary Cells and Isolated Mouse Gonadotropes

**Figure 8:**
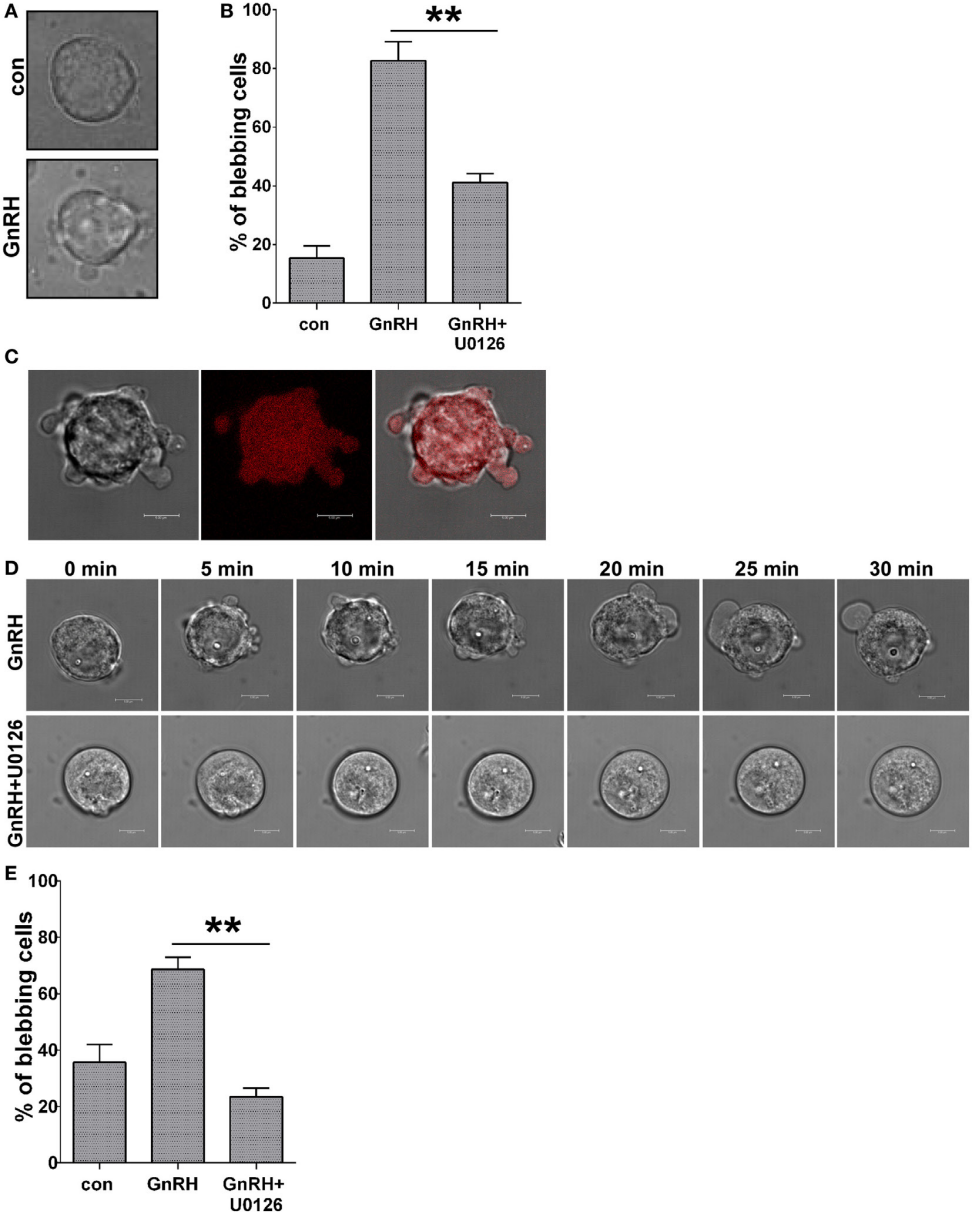
**GnRH induces ERK1/2-dependent bleb formation in primary cultures of rat pituitary cells and isolated mouse gonadotropes**. **(A)** DIC images from a confocal microscope of dissociated rat pituitary cells. Culture rat pituitary cells were prepared, and gonadotropes were identified by their Ca^2+^ response as described in Section “Materials and Methods.” The cells were treated with GnRH (10 nM) for 30 min. **(B)** Quantitation of the percentage of blebbing cells from dissociated rat pituitary cells. The cells were pretreated with U0126 (10 µM) for 30 min, followed by GnRH (10 nM) for additional 30 min. Data are mean ± SEM from three experiments. **(C)** DIC and fluorescent images from a confocal microscope of FACS-purified primary gonadotrope cells from adult GRIC/Ai9 mice treated with GnRH (30 min, 10 nM). The *scale bar* is 5 µm. **(D)** Images from a confocal microscopy time-lapse movie of FACS-purified primary gonadotrope cells. The cells were pretreated with or without U0126 (25 µM) for 30 min, followed by GnRH (10 nM) for additional 30 min. The *scale bar* is 5 µm. **(E)** Quantitation of the percentage of blebbing cells from panel **(D)** is presented (at least 30 cells for each experiment) and data are mean ± SEM. **p-value ≤0.01.

The data shown above confirmed that GnRH induces ERK1/2-dependent bleb formation in LβT2 cells. To determine whether this effect is also evident in primary rat pituitary cells in culture, dissociated rat pituitary cells were prepared and the gonadotropes were identified by their intracellular Ca^2+^ response to GnRH. Addition of GnRH resulted in bleb formation (Figure 8A). Quantitation of the percentage of blebbing cells showed that GnRH treatment resulted in bleb formation and ERK1/2 inhibition by U0126 significantly reduced bleb formation by GnRH (Figure 8B). We then isolated mouse pituitary gonadotropes by the use of FACS-sorted cells from adult GRIC/Ai9 mice and kept them in culture (42, 43, 66, 67). Time-lapse confocal microscopy revealed that GnRH-induced bleb formation (Figures 8C–E). In addition, preincubation of the cells with the MEK inhibitor, U0126, abolished bleb formation induced by GnRH (Figures 8D,E). Quantitation of the percentage of blebbing cells confirmed that GnRH induced ERK1/2-dependent bleb formation in primary gonadotropes in culture (Figure 8E).

## Discussion

Following the fate of ERK1/2 in LβT2 cells transfected with GnRHR-mCherry and ERK2-GFP and treated with GnRH, we noticed bleb formation in the cells. The blebs appear within ~2 min at a turnover rate of ~2–3 blebs/min and last for at least 90 min. The formation of the blebs is GnRHR-dependent since the GnRH antagonist (cetrorelix acetate) abolished bleb formation. Interestingly, retreatment with GnRH (30 min) 6 h later resulted in a priming effect, which is defined as an increase in cells response to the second exposure to GnRH compared with the first (Figure 2). A priming effect of the LH response to GnRH has been observed (68). The mechanism of the priming effect is under investigation.

A “Funnel Paradox” exists, namely, how is signal specificity maintained, while most of the receptor tyrosine kinases (RTKs) (of the 90 tyrosine kinases, 58 are receptor type) and GPCRs (>800) act *via* MAPKs with different biological responses. The most likely explanation is the presence of scaffold proteins and signaling complexes (signalosomes) (69, 70) that bring together different MAPK cascade members and their substrates and target them to specific sites in a spatio/temporal fashion (71, 72). Indeed, a signaling platform for ERK1/2 activation by GnRH including the GnRHR, c-Raf kinase, Ca^2+^-calmodulin, and ERK1/2 that was localized to low-density membrane microdomains (lipid rafts) has been proposed (29). Another complex including FAK and c-Src at FAs has been reported to be involved in ERK1/2 activation by GnRH in HEK 293 cells stably expressing the GnRHR (30).

In search of c-Src-interacting proteins, we came upon a large protein–protein complex associated with the GnRHR, a signalosome (33). The presence of FAK, paxillin, vinculin (residence of FAs), and tubulin led us to suggest that the signalosome resides in microtubules at the boundaries of FAs (33). We have shown that the role of the signalosome is to sequester a pool of GnRH-activated ERK1/2 in the cytosol for the phosphorylation of FAK and paxillin at FAs to mediate cell migration as recently proposed for GnRH-stimulated gonadotropes (34, 35). It is thought that RTK and GPCR ligands induce translocation of ERK1/2 to the nucleus to phosphorylate and activate transcription factors (10, 11). In the present work, we found a link between the signalosome and the blebs, suggesting that as with the signalosome, the blebs may be involved in cell migration.

ERK1/2 accumulated in the blebs, and we assume that ERK1/2 migrated from the signalosome to the blebs, since both are associated with an active membrane pool of ERK1/2. In support of this notion is the observation that various members of the signalosome were also found in the blebs. Since the signalosome is preformed and unlike the blebs is not dependent on GnRH (33), members of the signalosome were most likely recruited to the blebs. Also, formation of the blebs requires active ERK1/2 as evident by the use of the MEK1/2 inhibitor, U0126, which abolished bleb formation (Figures 3D–E). Interestingly, the use of the MEK1/2 inhibitor, U0126, which abolished bleb formation abolished also cell migration (data not shown). However, since the MEK inhibitor is not a specific bleb inhibitor, further studies are required to link bleb formation to gonadotrope migration.

Epidermal growth factor is a member of a family of peptide growth factors that activates the EGF receptors (EGFR). EGFR signaling pathway plays important roles in proliferation, differentiation, and migration of a variety of cell types, especially in epithelial cells (46). In addition, EGF is known to stimulate the ERK1/2 signaling pathway (46). Although the following ligands: EGF > GnRH > PMA > cAMP stimulate ERK1/2 in LβT2 cells, they produced little or no effect on bleb formation as compared to the robust effect of GnRH (GnRH > PMA > cAMP > EGF). The results indicate that ERK1/2 is required but not sufficient for bleb formation possibly due to compartmentalization of ERK1/2 in a ligand-dependent manner (Figures 4A–E).

The Rho family members RhoA, Rac1, and Cdc42 are small GTPases known to regulate actin cytoskeleton rearrangements. Godoy at el. (35) showed that GnRH inhibits p250RhoGAP expression in LβT2 cells. Hence, GnRH activates Rho family members, induces cytoskeletal rearrangements, and increases cell motility (35). The contractility for bleb retraction is provided by signaling through RhoA–ROCK–myosin. In this cascade, RhoA-GTP activates its effector kinase ROCK that directly phosphorylates myosin light chain, which then induces actomyosin contraction (38, 41). We therefore used the ROCK inhibitor Y27632 to examine its involvement in bleb formation. Indeed, Y27632 abolished bleb formation implicating the RhoA/ROCK signal in the process (Figures 4F–H).

GnRH receptor, c-Src, ERK1/2, FAK, paxillin, and tubulin, members of the above mentioned signalosome (33), accumulated in the blebs. On the other hand, vinculin was not present in the initial bleb expansion and was detected in the static phase. In addition, we could detect actin only at the steady phase of the blebs and during the retraction. Since vinculin is a known actin-binding protein, we assumed that vinculin and actin are involved in the static phase and in GnRH-induced bleb retraction, as indeed was the case. In addition, the activated ERK1/2 can phosphorylate myosin light chain kinase in a c-Src–FAK-dependent manner to further increase actomyosin contractility, which regulates adhesion disassembly and promote cell migration (73). Also, c-Src to FAK signaling and phosphorylation of FAK and paxillin *via* the activated ERK1/2, as observed in the signalosome (33), lead to FAs turnover at the cell front and cell migration (73–75), hence more blebs, supporting the signalosome–bleb pathway.

A major member of the signalosome is c-Src (33), and we have shown previously that GnRH activates c-Src and ERK1/2 activation is c-Src dependent (20). By virtue of its modular SH1, SH2, and SH3 domains, the soluble tyrosine kinase can act as scaffold for diverse signaling proteins (51). Time-lapse confocal microscopy identified c-Src in the blebs during expansion and retraction of the blebs (Figures 5A,B). Surprisingly, inhibition of c-Src activity by PP2 (Figure 5C) had no effect on bleb formation, suggesting that unlike ERK1/2, active c-Src is not required for bleb formation and that unlike the signalosome inactive c-Src is present in the blebs. Furthermore, since GnRH activates ERK1/2 *via* active c-Src (20), it is possible that active ERK1/2 migrated to the blebs from the signalosome or other cellular compartment and there is no further activation of ERK1/2 by GnRH *via* c-Src in the blebs. Alternatively, as we have previously shown, GnRH can also activate ERK1/2 in a c-Src-independent fashion (20) and this pool of ERK1/2 may reside in the blebs.

Previous studies have shown that GnRH stimulates remodeling of the cytoskeleton in gonadotrope-derived cell lines, primary cultures of dissociated pituitary cells (ovine and murine) and intact living pituitary that leads to the formation of lamellipodia and filopodia and increased cell migration (30, 34, 65). In addition, GnRH signaling to ERK requires actin polymerization (30, 34) and ERK inhibition did not inhibit the formation of lamellipodia and filopodia by GnRH (65). Common to the above studies is that the dynamic remodeling of the actin cytoskeleton was upstream to ERK1/2 activation in the GnRHR signaling network. On the other hand, in another system, activation of the ERK signaling pathway was required for the induction of actin polymerization and subsequent lamellipodium formation (76).

Our observations reported here differ from the above in particular in terms of signaling from the GnRHR to ERK1/2 and the blebs. Here, we show that GnRH induces blebs formation, which differ from lamellipodia or filopodia which are dependent on polymerizing actin filaments (36), while blebs growth is pressure driven, and not due to actin polymerization. In addition, we have shown that ERK1/2 inhibition strongly inhibits GnRH-induced bleb formation and cell migration. Hence, we propose that blebs formation is downstream to ERK1/2 activation in GnRHR signaling.

We emphasize here the signalosome–blebs pathway, suggesting that both are involved in cell migration. This is based on several lines of evidence; members of the signalosome are also found in the blebs; we have proposed that the role of the signalosome is to sequester a pool of active ERK1/2 to phosphorylate and activate FAK and paxillin at FAs to mediate cell migration (33) as shown for GnRH-stimulated gonadotropes (34, 35). Here, we show that bleb formation is dependent on active ERK1/2; hence, the potential link to the signalosome as a provider of active ERK1/2 in the vicinity of the membrane. Assuming that members of the signalosome migrated to the blebs, it is not clear if they migrated as a multi-protein complex, or separately. In support of the second assumption is the observation that some proteins migrated during bleb formation (GnRHR, c-Src, ERK1/2, FAK, and paxillin) and some during bleb stabilization and retraction (vinculin). The results lend support to the notion that the signalosome members were recruited separately to the blebs. In addition, although actin was present in the blebs, we could not detect actin in the signalosome (33), suggesting that blebs member’s proteins are not restricted to those found in the signalosome.

Importantly, we have confirmed that the blebs are formed in a more accurate physiological setting, as they were seen in cultured primary rat pituitary cells, in which the gonadotropes (5–10% of pituitary cells) (Figures 8A,B) were identified by their intracellular Ca^2+^ response to GnRH. They were also apparent in cultured FACS-sorted mouse primary gonadotropes from adult GRIC/Ai9 mice (Figures 8C–E). (42, 43, 66, 67) and in both cell models; we could demonstrate that the GnRH-induced bleb formation was dependent on active ERK1/2. We have thus established that this is a normal response of the gonadotropes to GnRH.

## Ethics Statement

Ethical consideration were approved for the use of animals in this study as stated in the text.

## Author Contributions

LN carried most of the experiments, and AT, AF, and MT carried some of the experiments. PM, SS, UB, and RS participated in the design of the experiments. ZN participated in the design of the experiments and in the preparation of the manuscript.

## Conflict of Interest Statement

The authors declare that the research was conducted in the absence of any commercial or financial relationships that could be construed as a potential conflict of interest.

## Supplementary Material

Video S1 **GnRH induces bleb formation in LβT2 cells, and GnRH receptor is present in the blebs**. Time-lapse movie of serum-starved LβT2 gonadotrope cells treated with GnRH (10 nM) (0–30 min). The treatment resulted in bleb formation.

Video S2 **ERK1/2 is present and involved in bleb formation**. Time-lapse movie of serum-starved LβT2 cells transfected with GnRH receptor-mCherry and ERK-GFP and treated with GnRH (0–15 min, 10 nM). GnRH stimulated bleb formation, while ERK1/2 accumulated in the blebs.
