# Supplementary material for: GnRH Induces ERK-Dependent Bleb Formation in Gonadotrope Cells, Involving Recruitment of Members of a GnRH Receptor-Associated Signalosome to the Blebs
Source: Front Endocrinol (Lausanne). 2017 Jun 2;8:113. doi: 10.3389/fendo.2017.00113 (PMC5454083; doi:10.3389/fendo.2017.00113)
Supplement: Supplementary file 3 [file Data_Sheet_1.PDF]

GnRH induces bleb formation in gonadotrope cells, involving recruitment of members of a GnRH receptor-associated signalosome to the blebs

Liat Rahamim-Ben Navi[1], Anna Tsukerman[2], Alona Feldman[2], Philippa Melamed[2], Melanija Tomić[3], Stanko S. Stojilkovic[3], Ulrich Boehm[4], Rony Seger[5], and Zvi Naor[1+]*

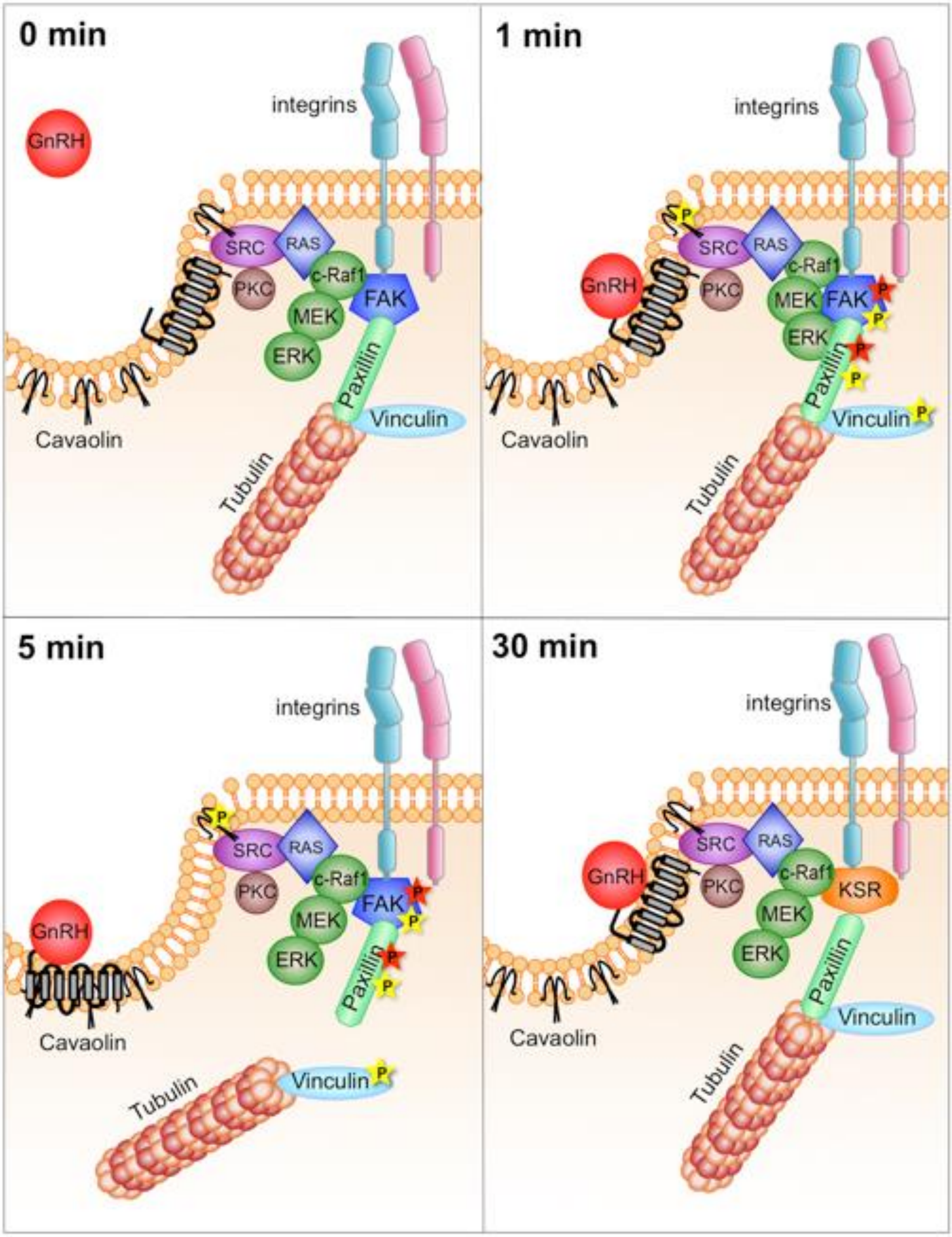

Fig 1. The signaling complex (Signalosome) is preformed, and exposure of LβT2 gonadotrope cells to GnRH induces a dynamic rearrangement. The signaling complex includes c-Src, protein kinase C, Ras, MAPK kinase 1/2, ERK1/2, tubulin, focal adhesion kinase (FAK), paxillin, vinculin, caveolin-1, kinase suppressor of Ras-1, and the GnRHR. Exposure to GnRH (1 min) causes ERK activation in a c-Src-dependent manner. The activated ERK1/2 subsequently phosphorylates FAK and paxillin. In parallel, caveolin-1, FAK, vinculin, and paxillin are phosphorylated on Tyr residues by GnRH-activated c-Src. 5 min after exposure to GnRH, MAPK kinase 1/2, ERK1/2, tubulin, vinculin, and the GnRHR detached from c-Src, but they reassociate within 30 min. On the other hand, FAK, paxillin, the protein kinase Cs, and caveolin-1 stay bound to c-Src, whereas kinase suppressor of Ras-1 appears in the complex only 30 min after GnRH stimulation.
